# Assessment of the Nutritional Composition, Antimicrobial Potential, Anticoccidial, and Antioxidant Activities of *Arthospira platensis* in Broilers

**DOI:** 10.3390/biology14040379

**Published:** 2025-04-07

**Authors:** Said Dahmouni, Zineb Bengharbi, Djilali Benabdelmoumene, Nardjess Benamar, Wasim S. M. Qadi, Esraa Adnan Dawoud Dawoud, Ebtesam Al-Olayan, Omar Dahimi, Andres Moreno, Mohd Asraf Mohd Zainudin, Ahmed Mediani

**Affiliations:** 1 Laboratory of Applied Animal Physiology, SNV Faculty, University of Mostaganem, Mostaganem 27000, Algeria; said.dahmouni@univ-mosta.dz (S.D.); zineb.bengherbi@univ-mosta.dz (Z.B.); djilali.benabdelmoumene@univ-mosta.dz (D.B.); nardjess.benamer@univ-mosta.dz (N.B.); omar.dahimi.etu@univ-mosta.dz (O.D.); 2 Institute of Systems Biology (INBIOSIS), Universiti Kebangsaan Malaysia (UKM), Bangi 43600, Malaysia; qadiwasim@gmail.com; 3 Faculty of Pharmacy, Universiti Kebangsaan Malaysia, Bangi 43650, Malaysia; p113229@ukm.edu.my; 4 Department of Zoology, College of Science, King Saud University, Riyadh 11451, Saudi Arabia; eo-layan@ksu.edu.sa; 5 Facultad de CC y TT Químicas, Universidad de Castilla-La Mancha, 13001 Ciudad Real, Spain; andres.moreno@uclm.es; 6 Faculty of Chemical Engineering & Technology, UniCITI Alam Campus, Universiti Malaysia Perlis (UniMAP), Sungai Chuchuh, Padang Besar 02100, Malaysia

**Keywords:** *Arthrospira platensis*, broiler nutraceuticals, bioactive compounds, antioxidants, anticoccidial, antibacterial activities

## Abstract

This study evaluates the chemical composition, fatty acid profiles, and bioactive properties of ethanolic (SPE), methanolic (SPM), and acetonic (SPA) extracts of *Arthrospira platensis*. The chemical analysis revealed a high protein content (72.08%), moderate lipid levels (6.49%), and a diverse fatty acid profile, dominated by polyunsaturated fatty acids. Antibacterial testing showed that SPE exhibited the strongest activity against Gram-negative bacteria, including *Escherichia coli* (ATCC 25922), *Enterobacter cloacae* (ATCC 49141), *Proteus mirabilis* (ATCC 25933), *Salmonella typhi* (ATCC 6539), *Salmonella* typhimurium (ATCC 14028), *Salmonella enteritidis* (ATCC 13076), *Salmonella gallinarum* (ATCC 9184), and *Pseudomonas aeruginosa* (ATCC 27853). The anticoccidial activity was also significant, with SPE reducing *Eimeria* sp. oocyst counts in poultry intestines and ceca. Antioxidant activity was highest in SPM, which also had the highest phenolic content. LC-MS/MS profiling of the methanolic extract revealed various bioactive compounds, including phenolic acids, flavonoids, carotenoids, chlorophylls, and phycobiliproteins. These results suggest that *A. platensis* has great potential as a nutraceutical supplement in poultry farming, offering a sustainable alternative to synthetic additives.

## Simple Summary

This study examines the chemical composition, bioactive properties, and health benefits of ethanolic, methanolic, and acetonic extracts of *Arthrospira platensis* (*Spirulina*). The extracts were found to have high protein content, moderate lipids, and beneficial fatty acids. They also demonstrated strong antibacterial, antioxidant, and anticoccidial activities. The ethanolic extract was particularly effective in inhibiting bacteria and reducing intestinal parasites in poultry. The study suggests that *Arthrospira platensis* could serve as a valuable natural supplement for improving poultry health and productivity.

## 1. Introduction

*Arthrospira* is a genus of cyanobacteria comprising several species, though only a few, such as *Arthrospira platensis* and *Arthrospira maxima*, are widely recognized as safe for human and animal consumption. Of the approximately 15 identified species, only these are edible and commercially cultivated due to their high nutritional and therapeutic potential [1]. *A. platensis*, in particular, is rich in bioactive compounds and offers benefits such as antibacterial and anticoccidial properties, making it a valuable natural supplement in various applications [2]. The interest in *Arthrospira* has surged due to its high content of protein, essential fatty acids, vitamins, minerals, and bioactive peptides that contribute to its health-promoting effects [3]. Among its key bioactive components, phycocyanin, polysaccharides, and phenolic compounds have been shown to possess strong antibacterial properties, establishing *Arthrospira* as an effective natural alternative to synthetic antibiotics [4,5]. Moreover, it provides significant benefits to broilers by improving growth performance, feed efficiency, and meat quality.

*A. platensis* supplementation in broiler production enhances immune function [6], reduces disease incidence, and promotes healthier gut microbiota, thereby improving digestion and nutrient absorption [7,8]. This is particularly valuable given the significant challenges posed by bacterial infections and parasitic diseases, such as coccidiosis, which adversely impact growth performance, feed efficiency, and overall health in broilers [9,10]. Additionally, *Arthrospira* offers anti-inflammatory and antioxidant properties, minimizing oxidative stress and enhancing overall broiler health [11], making it an especially valuable natural supplement under intensive farming conditions [4,12]. While antibiotics and chemical anticoccidial agents have traditionally been used to manage these issues, growing concerns about antibiotic resistance and drug residues have prompted the search for safer, natural alternatives [12,13]. Recent studies have demonstrated that *Arthrospira* exhibits broad-spectrum antibacterial activity against pathogens such as *Escherichia coli*, *Salmonella* spp., and *Staphylococcus aureus*, which are commonly associated with poultry diseases [14,15,16]. This antibacterial effect is primarily attributed to the presence of phycocyanin and phenolic compounds which disrupt bacterial cell membranes, inhibit bacterial growth, and enhance gut health [17,18].

Moreover, *Arthrospira* has demonstrated promising anticoccidial properties in broilers, effectively reducing oocyst shedding and enhancing gut integrity when included in poultry diets [19]. Its supplementation has been associated with improved immune responses, increased antioxidant activity, and enhanced growth performance, establishing it as a potent natural agent for controlling coccidiosis [20,21]. Studies have shown that *Arthrospira* extracts, particularly phycocyanin, can disrupt the life cycle of *Eimeria* spp., the causative parasite of coccidiosis, thereby reducing disease severity and promoting healthier gut flora in broilers [22,23]. In addition to its direct antibacterial and anticoccidial effects, *Arthrospira* serves as a prebiotic, enhancing the growth of beneficial gut microbiota such as *Lactobacillus* species, which further strengthens the bird’s resistance to infections [14]. This dual action not only improves the health and productivity of broilers but also enhances feed conversion ratios and meat quality [10,24].

The integration of *Arthrospira* into poultry feed offers a sustainable and eco-friendly approach to managing bacterial infections and coccidiosis, reducing dependence on conventional antibiotics and anticoccidial drugs [25,26]. This aligns with the growing demand for antibiotic-free poultry products and supports the development of safer, healthier, and more efficient poultry production systems [5,9]. This study aims to comprehensively analyze the chemical composition of *A. platensis*, complemented by LC-MS/MS profiling of its bioactive components, including phenolic compounds, flavonoids, carotenoids, phycobiliproteins, and chlorophyll derivatives. The antioxidant potential of *A. platensis* is assessed through 2,2′-azino-bis-(3-ethylbenzothiazoline-6-sulfonic acid) ABTS, ferric reducing antioxidant power (FRAP), and 2,2-diphenyl-1-picrylhydrazyl (DPPH) assays to explore its value as a functional food and natural therapeutic agent in broiler production. Additionally, the study further evaluates the antibacterial effects of *A. platensis* extracts (ethanolic, methanolic, and acetonic) against key commensal and pathogenic strains in broilers as well as its anticoccidial activity against *Eimeria* sp.

## 2. Materials and Methods

### 2.1. Samples Collections

Dried *Arthospira platensis* powder (CAS No. 724424-92-4) was obtained from a certified supplier. The powder was sieved to a fine consistency, with particles passing through a 60-mesh screen, and stored in airtight containers.

### 2.2. Nutritional Composition Arthospira platensis Powder

#### 2.2.1. Total Lipids and Fatty Acid Composition of *Arthospira platensis*

The fatty acid composition of *A. platensis* was determined following lipid extraction using the method of Markou et al. [27]. A 25 mg sample of dried *A. platensis* biomass was subjected to extraction with 3 mL of chloroform/methanol (2:1, *v*/*v*) as the initial solvent. To facilitate phase separation and isolate the organic fraction, an aqueous buffer (0.2 M sodium phosphate) was added, followed by an additional 2 mL of chloroform. The resulting organic phase containing the total lipids, was carefully collected and pooled for subsequent processing. The aliquots of the lipid extract were then esterified using BF_3_-methanol. The fatty acid composition of each aliquot was analyzed using gas chromatography (GC) on a Hewlett-Packard 6890 GC (Palo Alto, CA, USA) equipped with a 60 m fused silica capillary column (CP Sil 88, 0.20 mm internal diameter) and a flame ionization detector (FID). Helium was used as the carrier gas, and nitrogen was used as the make-up gas. The GC conditions were as follows: injection port temperature set at 200 °C, detector temperature at 250 °C, and oven temperature program starting at 150 °C (held for 3 min), then increased to 160 °C at 1.5 °C/min and held for 3 min, further increased to 190 °C at 1.5 °C/min and held for 1 min, and finally raised to 220 °C at 1 °C/1 min. A computer integrator was used to calculate retention times and percent peak areas. Fatty acids were identified by comparing sample retention times with those of standard fatty acids, including saturated fatty acids (SFAs), monounsaturated fatty acids (MUFAs), and polyunsaturated fatty acids (PUFAs) obtained from Sigma and Polyscience (St. Louis, Missouri, USA). Quantification was performed by normalizing and converting the area percentages to milligrams per 100 g of dry weight, using the lipid conversion factor.

#### 2.2.2. Total Protein

The crude protein content in *A. platensis* was estimated using the Kjeldahl method [28]. The protein content (%) was calculated using the formula:

$$Proteincontent\;(\%)=\frac{X\times 0.14\times V\times 6.25\times 100}{1000\times V1\times W}$$

where:

X = Titer value,

V = Total volume of the digest,

V1 = Volume of the digest used for distillation,

W = Weight of the sample used for digestion.

The constant 0.14 accounts for the nitrogen equivalent (1 mL of 0.01 N H_2_SO_4_~0.00014 g nitrogen), and the conversion factor of 6.25 is based on the assumption that nitrogen constitutes approximately 16% of most proteins.

#### 2.2.3. Moisture and Ash Determination

The moisture content of *A. platensis* powder was determined by drying the sample at 105 °C until a constant weight was achieved [28]. In this experiment, two grams (W2) of *A. platensis* were placed in pre-weighed Petri dishes (W1) and dried in an oven at 105 °C for 4 h. After cooling in a desiccator for 15 min, the final weight (W3) was recorded. The moisture content (%) was calculated using the formula:

$$Moisturecontent\;(\%)=\left(\frac{W2-W3}{W2-W1}\right)\times 100$$

where W1 is the weight of the empty dried Petri dishes, W2 is the weight of the samples, and W3 is the final weight of the samples and dishes.

Ash content, measured by incinerating the sample at 550 °C, indicates the mineral content of *A. Platensis*. Approximately 5 g of *A. platensis* (W1) were placed in crucibles (W2) and ashed in a muffle furnace at 550 °C until a light grey ash and constant weight were achieved. After cooling in desiccators, the samples were reweighed (W3). The ash content (%) was calculated using the formula:

$$Ashcontent\;(\%)=\frac{W3-W2}{W1}\times 100$$

where W1: The weight of the samples. W2: The weight of the crucibles. W3: The weight of the crucible with the ash

### 2.3. Preparation and Extraction of Arthospira platensis

Ethanol (99.9%), methanol (99.9%), and acetone (99.9%) were used to prepare the ethanolic (SPE), methanolic (SPM), and acetonic (SPA) of *A. platensis* extracts, respectively. A total of 50 g of *A. platensis* powder was macerated in 500 mL of each solvent for 72 h at room temperature [29]. The mixtures were left standing at room temperature and stirred every 24 h to ensure thorough extraction. After 72 h, the extracts were filtered, concentrated using a rotary evaporator (Buchi R-300) (Büchi Labortechnik AG, Flawil, Switzerland) at 40 °C, and stored at 4 °C.

### 2.4. Activity Testing of Arthospira platensis Against Bacterial and Coccidial Strains

#### 2.4.1. Antibacterial Activity Assessment

Ten bacterial strains representing a broad spectrum of Gram-positive and Gram-negative bacteria were selected for this study. The Gram-positive strains included *Listeria monocytogenes* (ATCC 19115) and *Staphylococcus aureus* (ATCC 25923). The Gram-negative strains included *Escherichia coli* (ATCC 25922), *Enterobacter cloacae* (ATCC 49141), *Proteus mirabilis* (ATCC 25933), *Salmonella typhi* (ATCC 6539), *Salmonella typhimurium* (ATCC 14028), *Salmonella enteritidis* (ATCC 13076), *Salmonella gallinarum* (ATCC 9184), and *Pseudomonas aeruginosa* (ATCC 27853). These strains were cultured on nutrient agar plates at 37 °C for 24 h to ensure optimal growth. The resulting bacterial suspensions were standardized to a concentration of approximately 1 × 10⁶ CFU/mL according to the 0.5 McFarland standard for consistent inoculum density. To prepare the antibacterial test solutions, *A. platensis* extracts were dissolved in 10% dimethyl sulfoxide (DMSO) to achieve a final concentration of 20 mg/mL. This solvent was selected to ensure maximum solubility and stability of the bioactive compounds. The antibacterial potential of the *A. platensis* extracts was evaluated using the agar well diffusion method as described by Balouiri et al. [30]. Mueller–Hinton agar plates were inoculated with 100 µL of each standardized bacterial suspension. Wells of 6 mm diameter were carefully cut into the agar medium and filled with 100 µL of the 20 mg/mL *A. platensis* extract solution. The plates were subsequently incubated at 37 °C for 24 h. Following incubation, the diameter of inhibition zones surrounding each well was measured to determine the antibacterial efficacy of the extracts.

#### 2.4.2. Anticoccidial Assay

Fecal samples from the intestines and ceca of 45-day-old ISA Hubbard broiler chickens were processed through sieving, centrifugation, and flotation in saturated sodium chloride (NaCl) solution. Purified oocysts were sporulated in 2.5% (*w*/*v*) potassium dichromate at room temperature for 48 h and stored at 4 °C [31]. Fecal oocyst counts from the intestines (IFC) and ceca (CFC) were performed using a McMaster chamber. For the anticoccidial assay, *Eimeria* oocysts (1 × 10^4^) were incubated with *A. platensis* extract at concentrations of 0.2, 0.5, and 1 mg/mL in 24-well plates at 28 °C for 48 h. The percentage of sporulation inhibition was calculated by counting sporulated and non-sporulated oocysts [32,33]. The lytic effect of extracts was assessed by measuring absorbance at 273 nm to quantify the release of cellular materials from lysed oocysts [34,35].

### 2.5. The Phytochemical Analysis of the Arthospira platensis Extract 

#### 2.5.1. Determination of Total Phenolic Content (TPC)

The TPC of *A. platensis* was measured using the Folin–Ciocalteu colorimetric method. To prepare the sample for analysis, a 1 mL aliquot of the *A. platensis* extract (1 mg/mL concentration) was mixed with 5 mL of Folin–Ciocalteu reagent (2 M), and pre-diluted at a 1:10 ratio with distilled water to reduce reagent concentration. The mixture was allowed to react at room temperature for 5 min. Following the initial incubation, 4 mL of a sodium carbonate solution (75 g/L) was added to neutralize the reaction and facilitate color development. The solution was then incubated at room temperature for 1 h in the dark to prevent photodegradation of the phenolic compounds. After incubation, the absorbance of the reaction mixture was measured at 765 nm using a Jenway 6715 spectrophotometer, with distilled water used as a blank to account for any background absorbance. A standard calibration curve was generated using gallic acid solutions with concentrations ranging from 0 to 100 µg/mL. The TPC in the *A. platensis* extract was calculated and expressed as milligrams of gallic acid equivalents per gram of crude extract (mg GAE/g), allowing for standardized reporting and comparison of results across studies (as described by Singleton and Rossi, with adaptations by Miliauskas and Van Beek [36]).

#### 2.5.2. Determination of Total Flavonoid Content (TFC)

The TFC of the extract was quantified using the colorimetric aluminum chloride (AlCl_3_) method, as described by Iqbal et al. [37]. A 0.75 mL aliquot of a 2% AlCl_3_ solution in methanol was combined with an equal volume (0.75 mL) of the extract. The mixture was thoroughly mixed and then incubated in the dark at room temperature for 10 min to ensure a full reaction between AlCl_3_ and the flavonoids present in the extract. Following incubation, the absorbance of the reaction mixture was measured at 430 nm using a spectrophotometer, with a blank sample used to correct for any baseline absorbance. A standard calibration curve was prepared using quercetin, with concentrations ranging from 0 to 100 µg/mL, allowing for precise quantification of flavonoid content in the extract. The total flavonoid content was expressed as milligrams of quercetin equivalents per gram of dry matter (mg QE/g), facilitating standardized reporting for comparative analysis across samples and studies.

#### 2.5.3. Quantification of Condensed Tannin Content

The condensed tannin content was determined following a modified method by Bouhalla et al. [38]. A 400 µL aliquot of the sample extract was mixed with 3 mL of a 4% vanillin solution in methanol, followed by the addition of 1.5 mL of concentrated hydrochloric acid. The mixture was then incubated at room temperature for 15 min to allow the reaction to proceed. After incubation, the absorbance was measured at 550 nm using a spectrophotometer. A standard curve was generated using catechin at concentrations ranging from 100 to 1000 µg/mL. The tannin content was expressed as milligrams of catechin equivalents per gram of crude extract (mg CE/g).

### 2.6. Determination of Antioxidant Activity

#### 2.6.1. DPPH Radical Scavenging Assay

The antioxidant activity of extracts from *A. platensis* was assessed using the DPPH radical scavenging assay, following the procedure described by Zakaria et al. [39]. A 50 µL aliquot of 5000 ppm extract was mixed with 5 mL of a 0.004% DPPH solution in methanol. The reaction mixture was incubated at 25 °C for 30 min to ensure adequate interaction between the extract and the DPPH radicals. Following incubation, the absorbance was measured at 517 nm to capture the characteristic DPPH absorption peak. A blank sample, consisting of 5 mL of DPPH solution without the extract, was used for the calibration. The percentage of DPPH radical inhibition was calculated using the following formula:

$$DPPH\;activity\%=\frac{A_{517}\;blank-A_{517}\;Sample}{A_{517}\;blank}\times 100$$

where A_517_ blank represents the absorbance of the blank, and A_517_ sample represents the absorbance of the sample containing the extract.

#### 2.6.2. ABTS Radical Scavenging Assay

The ABTS assay was performed according to the method outlined by Zeng et al. [40]. The ABTS radical cation solution was prepared by mixing 7 mM ABTS and 2.45 mM potassium persulfate in a 1:1 ethanol-water solution (*v*/*v*). The mixture was incubated in the dark at 25 °C overnight. The resulting solution was then diluted with 50% ethanol to reach an absorbance of 0.700 ± 0.005 at 734 nm. For the radical scavenging activity (RSA) measurement, 2.5 mL of the diluted ABTS solution was mixed with 0.25 mL of the extract solution (0.15 mg/mL lignin in DMSO). The mixture was allowed to react at room temperature for 12 min to facilitate the interaction between the ABTS radicals and the extract. The absorbance was measured at 734 nm using a Hach Lange DR6000 spectrophotometer (Hach Lange GmbH, Düsseldorf, Germany). A standard calibration curve was constructed using six Trolox concentrations, ranging from 15 to 40 mg/L.

#### 2.6.3. Ferric Reducing Antioxidant Power (FRAP) Assay

The ferric reducing antioxidant power (FRAP) assay was conducted following the method of Musa et al. [41]. The FRAP reagent was freshly prepared by mixing 300 mM acetate buffer (pH 3.6), 10 mM 2,4,6 TPTZ (2, 4, 6-tripyridyl-s-triazine) solution dissolved in 40 mM Hydrochloric acid (HCl), and 20 mM iron (III) chloride (FeCl_3_) 6H_2_O solution in a 10:1:1 ratio (*v*/*v*/*v*). For each assay, 3 mL of the freshly prepared FRAP reagent was mixed with 0.2 mL of the sample extract. The mixture was incubated at 37 °C for 5 min to allow the reduction of ferric (Fe^3^⁺) to ferrous (Fe^2^⁺) ions. Following incubation, the absorbance was then measured at 593 nm using a reagent blank as the reference. The antioxidant capacity of the sample was quantified using a calibration curve prepared with a 1 mM ferrous sulfate (FeSO_4_·7H_2_O) standard. Results were expressed as millimoles of Fe^2^⁺ equivalents per gram of sample (mM Fe^2^⁺/g), providing a standardized measure of the extract’s reducing power and its potential as an antioxidant.

### 2.7. Phytochemical Analysis Using LCMS/MS

For LC-MS/MS profiling, 50 mg of powder was extracted in 70% methanol, sonicated for 30 min, filtered with a 0.22 µm cellulose syringe filter (Whatman, Maidstone, UK), and analyzed using a C18 column (4.6 mm × 150 mm, 5 µm particle size, Agilent, Santa Clara, California, USA). A gradient setting of mobile phase A (methanol/acetonitrile) and mobile phase B (0.1% formic acid in deionized water) was used as follows: 0–2 min B (80%), 2–8 min B (50%), 8–15 min B (20%), and 15–17 min B (80%). The flow rate was set at 0.3 mL/min at a column temperature of 25 °C. The liquid chromatography system used was an Agilent 1260 Infinity LC system equipped with a mass spectrometer from Agilent. The mass spectrum was set to screen molecular fragments from 0 to 2700 *m*/*z* at a collision energy of 20 eV. Parent compounds were identified by targeting specific bioactive compounds, including phenolic acids, flavonoids, and apocarotenoids. These targeted parent compounds were further confirmed with fragment comparison based on previous work conducted by Phinyo et al. [42].

### 2.8. Statistical Analysis

All experiments were conducted in triplicates, and data were analyzed using SPSS version 25 (IBM Corp., Armonk, NY, USA). The results were expressed as mean ± standard deviation, and differences between treatments were evaluated using one-way ANOVA with Tukey’s post hoc test at a significance level of *p* < 0.05. 

## 3. Results and Discussion

### 3.1. Nutritional Composition of Arthospira platensis

The analysis of the nutritional composition of *A. platensis* powder in this study reveals its remarkable nutrient density, with each component expressed as g per 100 g dry matter (DM). The powder exhibited a low moisture content (8.47 g/100 g DM) indicative of its minimal water presence, which enhances its stability and long-term storage potential in powdered form. Protein was the most abundant component, reaching 72.08 g/100 g DM, which is considerably high, positioning *A. platensis* as a potent protein source. The ash content (13.41 g/100 g DM) reflects its richness in essential minerals, including calcium and iron (Table 1). The protein concentration reported in this study aligns closely with previous findings [43,44], which consistently highlight the high protein content of *A. platensis*. In addition, the ash content corroborates observations by Sopandi and Rohmah [45], further substantiating the mineral-rich composition of this microalga. The fatty acid composition of *A. platensis* shows a high proportion of PUFAs (63.00 g/100 g DM) compared to monounsaturated fatty acids (MUFAs, 8.40 g/100 g DM) and SFAs (28.60 g/100 g DM). Oleic acid (C18:1 [n-9]) was the predominant MUFA, accounting for 4.2 g/100 g DM, while linoleic acid (C18:2 [n-6], 35.1 g/100 g DM) and gamma-linolenic acid (C18:3 [n-6], 5.2 g/100 g DM) were the primary PUFAs. Minor variations across studies, as observed in lipid profiles, could be attributed to differences in environmental and cultivation conditions, a common variability noted in microalgal species. This fatty acid distribution aligns with prior studies by Kurt et al. [46] and Dibeklioglu et al. [47], which similarly reported high MUFA levels in *A. platensis*. Differences in fatty acid profiles observed in this and other studies likely result from variations in cultivation practices, environmental conditions, and nutrient availability [45].

### 3.2. Antibacterial Activity of *Arthospira platensis* Extracts

The antibacterial activity of *A. platensis* extracts varied significantly (*p* < 0.05) across bacterial strains, with notable differences observed between Gram-positive and Gram-negative bacteria (Table 2). Among the tested extracts, the ethanolic extract (SPE) consistently exhibited the highest inhibition zones, indicating its superior capacity to extract bioactive compounds with antimicrobial potential, such as polyphenols and phycocyanin. Methanol extract (SPM) also displayed significant antibacterial activity, although it was typically less effective than ethanol. However, for some Gram-positive bacteria, such as *Listeria monocytogenes*, SPM exhibited a higher inhibition zone (14.34 mm) compared to ethanol extract (SPE) (12.11 mm), suggesting a strain-dependent variation in antibacterial efficacy. In contrast, the acetonic extract (SPA) showed the weakest antibacterial activity, likely due to the limited solubility of certain bioactive compounds in acetone.

*Enterobacter* sp. demonstrated the highest sensitivity to SPE (19.00 mm), followed by *S. typhi*, *S.* Typhimurium, and *P. mirabilis* (16.10 and 16.00 mm, respectively). Additionally, SPE was more effective against *E. coli* (12.20 mm) compared to SPM (10.80 mm) and SPA (6.32 mm). These antibacterial results align with a previous study by Kaushik et al. [48], who reported the potent activity of ethanol-based *Arthrospira* extracts against Gram-negative bacteria. Overall, the ethanolic extract demonstrated the strongest antibacterial activity across most Gram-negative strains. For Gram-positive bacteria, SPM exhibited the highest inhibition zone against *L. monocytogenes* (14.34 mm), followed by SPE (12.11 mm) and SPA (8.70 mm), indicating that methanol is particularly effective in solubilizing polar bioactive compounds targeting *L. monocytogenes* bacteria. Similarly, for *S. aureus*, SPE showed the greatest activity (15.50 mm), followed by SPM (13.55 mm) and SPA (10.00 mm). These results are consistent with findings by Abbas et al. [49], who observed methanol’s efficacy in extracting antibacterial proteins and flavonoids. In alignment with prior research, this study provides baseline information on the antimicrobial potential of *A. platensis* against common fish pathogens. For instance, Rathi Bhuvaneswari et al. [50], demonstrated the antibacterial activity of *Arthrospira (A. platensis)* platensis aqueous extracts (SPAE) against a range of fish pathogens, highlighting its potential as a natural antimicrobial agent. Similarly, Nayyef and Thalij [51] evaluated SPAE against various clinically significant Gram-positive (*S. aureus*, *S. pneumoniae*, and *Granulicatella adicans*) and Gram-negative bacteria (*Pseudomonas aeruginosa*, *Klebsiella pneumoniae*, *E. coli*, and *Sphingomonas paucimobilis*). Their results indicated the broad-spectrum antibacterial activity of SPAE, supporting the efficacy of *A. platensis* extracts against diverse bacterial strains. Focusing on antibacterial activity, various metabolites have demonstrated a broad spectrum of efficacy against multiple bacterial strains. These bioactive compounds include sinapinic acid, vanillic acid, p-coumaric acid, kaempferol, ferulic acid, genistein, and gamma-linolenic acid (GLA). Each of these metabolites exhibits distinct mechanisms of action, contributing to their ability to inhibit bacterial growth and activity [52].

These findings highlight that ethanol and methanol are both effective solvents for extracting different classes of antibacterial compounds in *A. platensis*. Ethanol generally demonstrated the strongest overall antibacterial activity, likely due to its ability to dissolve non-polar and semi-polar compounds such as polyphenols, alkaloids, and terpenoids, which are known to disrupt bacterial cell membranes [16]. Methanol’s selective activity points to its efficacy in extracting polar compounds. Conversely, acetone displayed limited antibacterial efficacy due to its limited solubility for many bioactive compounds [53].

### 3.3. Anticoccidial Efficacy of Arthospira platensis Extracts

The study evaluated the anticoccidial effects of *A. Platensis* crude extracts including SPE, SPM, and SPA on poultry, by assessing oocyst counts and optical density (OD) measurements at 273 nm (Table 3). In the untreated control group, oocyst counts were significantly elevated in both the intestines (32.33 × 10^2^ oocysts/g) and caeca (50.73 × 10^2^ oocysts/g), indicating the progression of infection. Among the tested extracts, SPE demonstrated the most substantial anticoccidial activity, reducing oocyst counts to 1.14 × 10^2^ oocysts/g in the intestines and 2.02×10^2^ oocysts/g in the caeca. This pronounced reduction can be attributed to the bioactive compounds present in *A. platensis*, such as phycocyanin and polysaccharides, which have been reported for their antiparasitic properties [54,55].

The higher OD value at 273 nm for SPE (2.92) supports the conclusion that SPE contains elevated concentrations of bioactive compounds, supporting its strong anticoccidial efficacy. SPM exhibited a moderate anticoccidial effect, reducing oocyst counts to 5.24 × 10^2^ oocysts/g in the intestines and 9.32 × 10^2^ oocysts/g in the caeca. While less effective than SPE, these reductions remain significant relative to the untreated group. The moderate efficacy of SPM can be attributed to differences in the solubility and concentration of bioactive compounds extracted by methanol. Methanol extracts of *A. Platensis* are recognized for their antimicrobial properties, which may explain the partial reduction in oocyst counts [56]. SPM’s OD at 273 nm (2.60) indicates a relatively lower concentration of bioactive compounds compared to SPE. In contrast, SPA exhibited the least anticoccidial activity, with oocyst counts (8.22 × 10^2^ oocysts/g in the intestines and 12.60 × 10^2^ oocysts/g in the caeca), suggesting limited efficacy. This reduced activity is likely due to acetone’s preferential extraction of non-polar compounds, which may exclude many of the bioactive components responsible for *A. Platensis*’s antiparasitic effects. This is reflected in SPA’s lower OD value (1.38), indicating a reduced concentration of active compounds compared to the other extracts [57].

The extracts of *A. Platensis*, particularly the SPE and SPM, exhibit considerable anticoccidial activity, evidenced by the marked reduction in oocyst counts in infected poultry. The enhanced efficacy of SPE can be attributed to its ability to extract a broader range of bioactive compounds, including phycocyanin and polysaccharides, known for their immunomodulatory and antiparasitic properties [52,58]. In contrast, SPA exhibited comparatively lower efficacy, likely due to acetone’s limited capacity to extract the full spectrum of bioactive compounds. These findings align with previous research on the antiparasitic potential of *Arthrospira*, supporting its application as a natural alternative to synthetic anticoccidial drugs in poultry production. This strategy may help mitigate the risks associated with chemical anticoccidials, such as the development of drug-resistant parasites and environmental contamination [59]. These findings reinforce *A. platensis*’s potential to replace synthetic antibiotics and anticoccidials, addressing challenges related to antimicrobial resistance and chemical toxicity. As highlighted by Khan et al. [9], the shift toward natural alternatives is critical for sustainable poultry production. Overall, the promising bioactivities of *A. platensis* make it a valuable candidate for functional feed, promoting animal health and enhancing productivity in broiler farming.

### 3.4. Total Phenolic, Flavonoid, and Tannin Content in Arthospira platensis Extracts

The comparative analysis of polyphenols, flavonoids, and tannins in SPE, SPM, and SPA extracts of *A. platensis* reveals significant differences in solvent extraction efficiency, reflecting variations in their polarity and affinity for bioactive compounds. SPM exhibited the highest total phenolic content (TPC) (18.90 mg gallic acid equivalent (GAE)/g of extract), highlighting methanol efficacy in extracting polar phenolic compounds. SPE demonstrated intermediate polyphenol levels (15.74 mg GAE/g), with its relatively lower polarity, yielded the least amount of polyphenols (13.50 mg GAE/g) (Table 4).

The total flavonoid content was comparable between SPE (9.07 mg quercetin equivalents per gram (QE)/g) and SPM (9.10 mg QE/g), reflecting similar polarities of these solvents and their effectiveness in flavonoid extraction. In contrast, SPA exhibited significantly lower flavonoid levels (6.83 mg QE/g), attributed to the limited solubility of flavonoids in less polar acetone solvents. Tannin content followed a similar trend, with SPM (2.82 mg TAE/g) and SPE (2.72 mg TAE/g) comparable yields, while SPA exhibited the lowest tannin concentration (2.0 mg TAE/g).

The results emphasize the significant effect of solvent choice on the extraction of phenolic, flavonoids, and tannin compounds from *A. platensis*. Methanol, due to its high polarity, was the most effective for extracting total phenolics, aligning with studies indicating methanol’s high efficacy for extracting both free and bound phenolic compounds due to its polarity [60]. Ethanol also yielded moderate phenolic content, while acetone had the lowest phenolic content, consistent with findings that less polar solvents, like acetone, are less effective for phenolic extraction [61]. Similarly, flavonoid content showed no significant difference between ethanol (9.07 mg QE/g) and methanol (9.10 mg QE/g) extracts, suggesting that both solvents have a similar effect for flavonoid extraction from *A. platensis* [62]. However, SPA had significantly lower flavonoid content (6.8 mg QE/g), likely due to the reduced solubility of flavonoids in acetone [63]. Tannin content varied slightly between ethanol (2.72 mg TAE/g) and methanol (2.82 mg TAE/g) extracts, while SPA had significantly lower levels (2.00 mg TAE/g), supporting research that polar solvents like ethanol and methanol are more suitable for tannin extraction [64]. In a previous study, the phytochemical analysis of *A. platensis* revealed its richness in bioactive compounds. The TPC was the highest among the studied extracts, measured at 8.59 mg GAE/g of extract. Similarly, *A. platensis* exhibited the highest flavonoid content, with a value of 22.70 mg rutin/g of extract. Additionally, the condensed tannin content was quantified at 3.01 mg CE/g of extract [65].

### 3.5. Antioxidant Activities of Arthospira platensis Extracts

The antioxidant activities of *A. platensis* extracts, as assessed by the 2,2-diphenyl-1-picrylhydrazyl (DPPH), ferric reducing antioxidant power (FRAP), and 2,2′-azino-bis-(3-ethylbenzothiazoline-6-sulfonic acid) ABTS assays, exhibited significant variation depending on the solvent used for extraction (Table 5). Among the tested solvents, the methanolic extract demonstrated the highest antioxidant activity across all assays. Specifically, the DPPH radical scavenging activity of the methanolic extract was 91.07%, the highest among the extracts, followed by acetone (77.51%) and ethanol (62.17%). In the FRAP assay, which measures the ferric reducing antioxidant power, methanol also outperformed the other solvents, with a value of 5.28 mmol Fe^2^⁺/g, significantly higher than the ethanol (3.07 mmol Fe^2^⁺/g) and acetone (3.31 mmol Fe^2^⁺/g) extracts. For the ABTS assay, the methanolic extract again exhibited the highest activity, with 5.81 mmol Trolox/g, followed by the ethanolic extract (4.94 mmol Trolox/g) and the acetone extract (3.27 mmol Trolox/g). These findings are consistent with previous studies. Sommella et al. [66] attributed the strong radical scavenging activity measured by DPPH to specific carotenoids and chlorophylls present in *A. platensis*. Shalaby and Shanab [67] reported that methanolic extracts of *A. platensis* contain phycobilin pigments (C-phycocyanin, allophycocyanin, and C-phycoerythrin) and bioactive compounds such as phenolics (e.g., pyrogallol) and flavonoids (e.g., catechin), which contribute to its potent antioxidant activity. Additionally, the reducing power of *A. platensis* extracts in FRAP assays has been reported by Golmakani et al. [68].

In the present study, the antioxidant properties of *A. platensis* extracts are attributed to the presence of alkaloids, tannins, flavonoids, and phenols, as reported by Chauhan et al. [69], which act as free radical scavengers. Similar studies on other blue-green algae (BGA) species have demonstrated the presence of phenolics, vitamins, and carotenoids, which are effective antioxidants and can be consumed as dietary supplements to mitigate oxidative stress and prevent health issues [59,70]. Moreover, other studies have reported comparable findings. For instance, ethanolic and aqueous extracts of *S. platensis* showed 96.33% radical scavenging activity, while *S. maxima* exhibited 25.73% [71]. Crude extracts of *S. platensis* displayed 45.75% radical scavenging activity when tested using the DPPH assay [16]. These findings collectively highlight the significant potential of *A. platensis* as a source of natural antioxidants.

These results highlight the superior antioxidant potential of the methanolic extract of *A. platensis*, particularly in its ability to neutralize free radicals and reduce metal ions. The lower antioxidant activity observed in the acetonic extract may be attributed to the reduced solubility of certain bioactive compounds in acetone, which are likely responsible for the observed antioxidant effects. The variation in antioxidant activity among the solvents aligns with broader findings in the field, emphasizing the pivotal role of solvent choice in extraction efficacy. Solvent polarity is a key factor influencing the solubility of phenolic acids, flavonoids, and other bioactive compounds known for their antioxidant properties. Methanol, with its higher polarity, proved to be more effective at solubilizing a wider range of polar antioxidant compounds. In contrast, acetone and ethanol, with lower polarities, exhibited more selective extraction capacities. This selective extraction likely resulted in reduced antioxidant activity, as fewer phenolic and flavonoid compounds were present in the extracts [72,73].

### 3.6. LC-MS/MS Profiling of Arthospira platensis Extracts

The LC-MS/MS profiling of the methanolic extract of *A. platensis*, selected for its superior antioxidant activity among the tested extracts, reveals the presence of diverse bioactive compounds, including phenolic acids, flavonoids, carotenoids, chlorophylls, and phycobiliproteins, each contributing to its potent health benefits (Table 6). Phenolic acids, such as caffeic and p-coumaric acid, exhibit strong antioxidant activity by scavenging free radicals, while flavonoids, including quercetin, rutin, and kaempferol, contribute to anti-inflammatory and immune-modulating properties. Phycocyanin, identified as a key phycobiliprotein, is known for its antioxidant, anti-inflammatory, and immune-boosting effects. Chlorophyll derivatives, including chlorophyll b and OH-chlorophyll a, enhance antioxidant capacity and contribute to detoxification processes. Additionally, the presence of α-tocopherol (vitamin E) further reinforces *A. platensis*’s potential for combating oxidative stress, enhancing its value as a functional food and nutraceutical ingredient.

The LC-MS/MS profiling of *A. platensis* revealed a rich composition of bioactive compounds. Compound 1, identified as caffeic acid (C_9_H_8_O_4_), exhibited a pseudo-molecular ion at *m*/*z* 179.034 [M−H]−, with characteristic fragments at *m*/*z* 135.034 [M−H−CO_2_]−, indicative of carboxyl group loss, along with an ion at *m*/*z* 119.045, consistent with caffeic acid fragmentation as reported by Tan et al. [74]. Compound 2, p-coumaric acid (C_7_H_6_O_4_), displayed a pseudo-molecular ion at *m*/*z* 153.018 [M−H]−, based on fragment ions at *m*/*z* 109.020 [M−H−CO_2_]− and *m*/*z* 91.019. This identification aligns with established spectrometric profiles of phenolic acids known for their antioxidant activity. Among the carotenoids, β-carotene (C_40_H_56_), assigned to compound 9, displayed a pseudo-molecular ion at *m*/*z* 569.395 [M−H]−, with fragments at *m*/*z* 551.387, 533.379, and 425.369, consistent with carotenoid fragmentation by sequential isoprene unit loss. Zeaxanthin (C_40_H_56_O_2_), compound 4, showed a pseudo-molecular ion at *m*/*z* 567.412 [M−H]−, with fragments at *m*/*z* 551.387 and 533.379, indicating its antioxidant potential, as both β-Carotene and Zeaxanthin are known for free radical scavenging. Chlorophyll derivatives were identified, further underscoring *A. platensis*’s antioxidant capacity. Compound 14 was identified as chlorophyll b (C_55_H_70_MgN_4_O_6_) with a pseudo-molecular ion at *m*/*z* 905.529 [M−H]− and fragments at *m*/*z* 627.424, 593.411, and 565.401. Compound 15, with a pseudo-molecular ion at *m*/*z* 893.504 [M−H]− and fragments at *m*/*z* 615.391, 581.377, and 553.367, was assigned as OH-chlorophyll a (C_55_H_72_O_6_N_4_Mg), highlighting hydroxylated chlorophyll derivatives’ role as antioxidants, corroborating similar identification in studies by Sommella et al. [66].

These findings support and extend work on *A. platensis*, confirming a wide range of bioactive compounds and emphasizing the presence of phenolic acids, carotenoids, and chlorophyll derivatives. The phenolic acids (e.g., caffeic acid, *m*/*z* 179.034; p-coumaric acid, *m*/*z* 153.018) contribute to antioxidant properties in *A. platensis* extracts, while carotenoids such as β-carotene (*m*/*z* 569.395) and Zeaxanthin (*m*/*z* 567.412) enhance its radical scavenging abilities [66]. Additionally, chlorophyll derivatives, such as chlorophyll b (*m*/*z* 905.529) and hydroxylated chlorophyll a (*m*/*z* 893.504), affirm *A. platensis*’s potent antioxidant profile, consistent with earlier studies.

## 4. Conclusions

This study underscores the potential of *A. platensis* as a natural therapeutic agent in broiler production. Its rich nutritional composition, including high protein content, essential fatty acids, and vital minerals, highlights its value as a dietary supplement. The ethanolic and methanolic extracts of *A. platensis* demonstrated significant antibacterial activity against both Gram-positive and Gram-negative bacteria, attributed to their high polyphenol and phycocyanin content. Furthermore, *A. platensis* exhibited strong anticoccidial effects, effectively reducing *Eimeria* sp. oocyst sporulation and inducing cell lysis. These findings position *A. platensis* as a sustainable and effective alternative to synthetic antibiotics and anticoccidials, promoting poultry health and productivity while addressing the increasing demand for natural and safe interventions in animal nutrition. Additionally, its robust antioxidant capacity, confirmed by ABTS, FRAP, and DPPH assays, highlights its potential to mitigate oxidative stress. The LC-MS/MS analysis of the methanolic extract, chosen for its superior antioxidant activity, revealed a diverse profile of bioactive compounds, including phenolic acids, flavonoids, carotenoids, chlorophylls, and phycobiliproteins, each contributing to its health-promoting properties. Together, these findings demonstrate the potential of *A. platensis* as a holistic solution in sustainable poultry farming and as a source of bioactive compounds with broad therapeutic applications.

## Figures and Tables

**Table 1:** Chemical composition and fatty acid profile of *Arthospira platensis* powder.

*Arthospira platensis* (g/100 g of Dry Matter)		
Moisture %	8.47 ± 0.88	
Protein %	72.08 ± 2.22	
Ash %	13.41 ± 0.22	
Lipid %	6.49 ± 0.86	
Fatty acids (%) profile	C14	1.00 ± 0.13
	C15	0.40 ± 0.02
	C16	16.20 ± 0.90
	C16:1 [n-7]	1.50 ± 0.01
	C17	1.20 ± 0.01
	C16:2 [n-6]	20.40 ± 1.08
	C18	2.80 ± 0.03
	C18:1 [n-13]	0.30 ± 0.01
	C18:1 [n-9]	4.20 ± 0.04
	C18:1 [n-7]	2.00 ± 0.01
	C18:2 [n-6]	35.10 ± 0.91
	C19	0.20 ± 0.001
	C18:3 [n-6]	5.20 ± 0.04
	C20	5.70 ± 0.08
	C20:1 [n-9]	0.40 ± 0.001
	C20:2	1.30 ± 0.02
	C20:3 [n-6]	0.40 ± 0.007
	C21	0.20 ± 0.01
	C20:4 [n-6]	0.60 ± 0.03
	C22	0.90 ± 0.04
	Total SFA	28.60 ± 0.91
	Total MUFA	8.40 ± 0.044
	Total PUFA	63.00 ± 1.41

Values are expressed as mean ± standard deviation (*n* = 3).

**Table 2:** Antibacterial activity of ethanol, methanol, and acetone extracts of *Arthospira platensis* against selected bacterial strains.

Bacteria Strains	SPE	SPM	SPA
Zone of inhibition (mm)			
*Escherichia coli*	12.20 ± 0.10 ^a^	10.80 ± 0.80 ^b^	6.32 ± 0.50 ^c^
*Enterobacter* sp.	19.00 ± 0.12 ^a^	15.22 ± 0.66 ^b^	7.00 ± 0.22 ^c^
*Salmonella typhi*	16.10 ± 0.63 ^a^	12.10 ± 1.10 ^c^	14.42 ± 0.74 ^b^
*Salmonella typhimurium*	16.00 ± 0.00 ^a^	12.60 ± 0.80 ^b^	10.70 ± 0.64 ^b^
*Salmonella Enteritidis*	10.12 ± 1.12 ^b^	13.00 ± 0.73 ^a^	9.00 ± 0.14 ^b^
*Salmonella gallinarum*	12.66 ± 0.84 ^a^	11.00 ± 0.52 ^b^	10.00 ± 0.15 ^c^
*Proteus mirabilis*	16.00 ± 0.11 ^a^	13.00 ± 050 ^b^	11.40 ± 0.22 ^c^
*Pseudomonas aeruginosa*	14.00 ± 0.43 ^a^	10.00 ± 0.08 ^b^	9.32 ± 0.50 ^b^
*Listeria monocytogenes*	12.11 ± 0.70 ^b^	14.34 ± 0.82 ^a^	8.70 ± 0.08 ^c^
*Staphylococcus aureus*	15.50 ± 0.82 ^a^	13.55 ± 0.80 ^b^	10.00 ± 0.55 ^c^

Values are expressed as mean ± standard deviation (*n* = 3). ^a–c^ Different superscript letters within the row indicate significant differences between solvent extractions at (*p* < 0.05). SPE: Crude Ethanol Extract, SPM: Crude Methanol Extract, SPA: Crude Acetone Extract. mm: millimeter.

**Table 3:** Anticoccidial efficacy of *Arthospira platensis* extracts on poultry: comparative analysis of oocyst reduction and bioactive compound absorption at 1 mg/mL.

Parameter	Initial Values	SPE	SPM	SPA
IFC (10^3^ oocysts/g)	32.33 ± 1.62 ^a^	1.14 ± 0.05 ^d^	5.24 ± 0.12 ^c^	8.22 ± 0.16 ^b^
CFC (10^3^ oocysts/g)	50.73 ± 0.90 ^a^	2.02 ± 0.01 ^e^	9.32 ± 0.42 ^c^	12.60 ± 0.50 ^b^
OD (IFC)	0.89 ± 0.004 ^d^	2.92 ± 0.001 ^a^	2.60 ± 0.001 ^b^	1.38 ± 0.006 ^c^
OD(CFC)	0.08 ± 0.001 ^e^	0.77 ± 0.004 ^a^	0.44 ± 0.005 ^c^	0.21 ± 0.001 ^d^

Values are expressed as mean ± standard deviation (*n* = 3). ^a–e^ Different superscript letters represent significant differences within the row (*p* < 0.05). IFC: Intestines Fecal Counts, CFC: Caeca Fecal counts, OD: Optical density. SPE: Crude Ethanol Extract, SPM: Crude Methanol Extract, SPA: Crude Acetone Extract.

**Table 4:** Total polyphenols, flavonoids, and tannin content in *Arthospira platensis* extracts using ethanol, methanol, and acetone as solvents.

Compound	SPE	SPM	SPA
Polyphenol (mg GAE/g)	15.74 ± 0.08 ^b^	18.90 ± 1.10 ^a^	13.50 ± 0.70 ^c^
Flavonoid (mg QE/g)	9.07 ± 0.05 ^a^	9.10 ± 0.72 ^a^	6.83 ± 0.42 ^b^
Tannin (mg CE/g)	2.72 ± 0.24 ^a^	2.82 ± 0.31 ^a^	2.00 ± 0.20 ^b^

Values are expressed as mean ± standard deviation (*n* = 3). ^a–c^ Different superscript letters represent significant differences within the row (*p* < 0.05). SPE: Crude Ethanol Extract, SPM: Crude Methanol Extract, SPA: Crude Acetone Extract. GAE/g: gallic acid equivalents per gram, QE/g: quercetin equivalents per gram, CE/g: catechin equivalents per gram.

**Table 5:** Comparison of antioxidant activities (ABTS, FRAP, and DPPH) of *Arthospira platensis* extracts.

Parameter	SPE	SPM	SPA
DPPH (%)	62.17 ± 5.51 ^c^	91.07 ± 7.44 ^a^	77.51 ± 6.28 ^b^
FRAP (mmol Fe^2^⁺/g extract)	3.07 ± 0.11 ^c^	5.28 ± 0.19 ^a^	3.31 ± 0.09 ^b^
ABTS (mmol Trolox/g)	4.94 ± 0.22 ^b^	5.81 ± 0.13 ^a^	3.27 ± 0.09 ^c^

Values are expressed as mean ± standard deviation (*n* = 3). ^a–c^ Different superscript letters represent significant differences within the row (*p* < 0.05). SPE: Crude Ethanol Extract, SPM: Crude Methanol Extract, SPA: Crude Acetone Extract. DPPH: 2,2-diphenyl-1-picrylhydrazyl, FRAP: ferric reducing antioxidant power, and ABTS: 2,2′-azino-bis-(3-ethylbenzothiazoline-6-sulfonic acid).

**Table 6:** LC-MS/MS mass spectrum profile of selected phenolic compounds, flavonoids, carotenoids, phycobiliproteins, and chlorophyll derivatives in methanolic *Arthospira platensis*.

Class	RT (min)	*m*/*z* (M − H)^−^	*m*/*z* (M + H)^+^	Molecular Formula	MS/MS Fragmentation Ions	Number	Proposed Compound
Phenolic Acid	5.4	179.034	181.050	C_9_H_8_O_4_	135.034, 119.045	1	Caffeic acid
Phenolic Acid	6.3	153.018	155.033	C_7_H_6_O_4_	109.020, 91.019	2	p-Coumaric acid
Flavonoid Glycoside	7.8	301.035	303.051	C_15_H_10_O_6_	285.029, 271.026, 255.020	3	Quercetin
Carotenoid	8.0	567.412	569.428	C_40_H_56_O_2_	551.387, 533.379, 515.360	4	Zeaxanthin
Flavonoid Glycoside	8.2	449.109	451.125	C_21_H_20_O_11_	287.055, 151.003	5	Rutin
Flavonoid Aglycone	9.0	271.061	273.077	C_15_H_10_O_5_	153.018, 125.015, 109.020	6	Kaempferol
Polyphenol	9.5	355.092	357.108	C_15_H_14_O_9_	193.034, 175.025, 121.021	7	Chlorogenic acid
Carotenoid	9.8	597.387	599.403	C_40_H_52_O_4_	581.371, 563.354, 545.344	8	Astaxanthin
Carotenoid	10.1	569.395	571.411	C_40_H_56_	551.387, 533.379, 425.369	9	β-carotene
Vitamin E (Tocopherol)	10.5	N.D	431.384	C_29_H_50_O_2_	413.368, 395.355, 377.347	10	α-Tocopherol
Carotenoid	11.2	565.371	567.387	C_40_H_52_O_2_	549.354, 531.345, 513.335	11	Canthaxanthin
Carotenoid	11.7	537.363	539.379	C_40_H_52_	519.354, 501.345, 437.335	12	Lycopene
Flavonoid Aglycone	12.0	285.040	287.055	C_15_H_10_O_7_	151.003, 125.015, 107.007	13	Myricetin
Chlorophyll	14.8	905.529	907.545	C_55_H_70_MgN_4_O_6_	627.424, 593.411, 565.401	14	Chlorophyll b
Chlorophyll Derivative	15.6	893.504	895.520	C_55_H_72_O_6_N_4_Mg	615.391, 581.377, 553.367	15	OH-Chlorophyll a
Phycobiliprotein	16.5	614.342	616.358	C_33_H_38_N_4_O_6_	598.324, 570.309, 500.279	16	Phycocyanin

LC-MS/MS: Liquid chromatography-tandem mass spectrometry, RT: Retention time, N.D; Not Detected.

## Data Availability

The data used to support the findings of this study are included within the article. Any other data can be available upon request.
